# Measuring the Unmeasurable: The Psychometrics of Life History Strategy

**DOI:** 10.1007/s12110-017-9307-x

**Published:** 2017-11-15

**Authors:** Stefan L. K. Gruijters, Bram P. I. Fleuren

**Affiliations:** 1 Department of Work and Social Psychology, Maastricht University, Maastricht, the Netherlands; 2 Faculty of Psychology and Educational Sciences, Open University of the Netherlands, Heerlen, the Netherlands

**Keywords:** Life history strategy, Ultimate-proximate distinction, Measurement models, Psychometrics, Formative models, Latent variables, Validity

## Abstract

Within evolutionary biology, life-history theory is used to explain cross-species differences in allocation strategies regarding reproduction, maturation, and survival. Behavioral scientists have recently begun to conceptualize such strategies as a within-species individual characteristic that is predictive of behavior. Although life history theory provides an important framework for behavioral scientists, the psychometric approach to life-history strategy measurement—as operationalized by K-factors—involves conceptual entanglements. We argue that current psychometric approaches attempting to identify K-factors are based on an unwarranted conflation of functional descriptions and proximate mechanisms—a conceptual mix-up that may generate unviable hypotheses and invites misinterpretation of empirical findings. The assumptions underlying generic psychometric methodology do not allow measurement of functionally defined variables; rather these methods are confined to Mayr’s proximate causal realm. We therefore conclude that K-factor scales lack validity, and that life history strategy cannot be identified with psychometrics as usual. To align theory with methodology, suggestions for alternative methods and new avenues are proposed.

Evolutionary biologists forwarded life-history (LH) theory to explain cross-species differences in allocation strategies with regard to reproduction, maturation, and survival (e.g., Stearns 1992). LH theory provides an evolutionary understanding of how species deal with the allocation of energetic resources. For any organism, a limited energetic “budget” has to be earmarked both for the development and maintenance of a well-adapted organism and for reproductive activities. Because these *reproductive* and *somatic* efforts are often mutually exclusive (e.g., time spent on growth of an organism delays reproduction), this situation creates a LH optimization problem (Schaffer 1983). That is, the LH problem entails that any increase of budget toward one fitness-relevant goal (e.g., growing) has to be met by a decrease of investment toward other fitness-enhancing activities (e.g., pubertal timing; Ellis 2004). Such investments involve fitness trade-offs (Garland 2014; Stearns 1989), and on both phylogenetic and ontogenetic levels solutions to the LH problem are given shape. The best-suited solution (or, LH strategy) arising in species during evolution (and in organisms during development) to optimize the LH problem is in turn contingent on ecological conditions.

In addition to its importance as an evolutionary biological model, LH theory has recently found application in evolutionary (approaches to) psychology (e.g., Buss 2009; Kaplan and Gangestad 2004). This extension of LH theory to psychology has been accompanied by a psychometric approach relying on self-report instruments. Figueredo and colleagues contributed extensively to the psychometric LH literature by developing and testing various LH strategy measurement instruments (e.g., Figueredo et al. 2005, 2006, 2007, 2013, 2014; Olderbak et al. 2014).

Proposed measurement instruments include the Arizona Life-History Battery (ALHB; Figueredo et al. 2007), the High-K strategy scale (HKSS; Giosan 2006), the mini-K (Figueredo et al. 2006), and the recently published K-SF-42 (Figueredo et al. 2017). These questionnaires are designed to measure the differential *K*-factor that has its roots in the work of Rushton (1985). Factor scores on such scales purportedly position individuals on a dimension of fast to slow LH strategies, with fast strategies indicating a psychological “orientation” toward increased reproductive efforts. These differential strategies are assumed to be reflected by individual differences in, for example, risk-taking tendencies, altruism and cooperation, and time preference (e.g., Figueredo et al. 2006). Although the K-factor exhibits considerable within-species heritable variation (Figueredo et al. 2004), individual differences are also thought to originate from the effects of early-life experiences, through mechanisms allowing for developmental plasticity (e.g., Frankenhuis and de Weerth 2013; Nettle and Bateson 2015; Nettle et al. 2010, 2013).

A central assumption in the psychometric work on LH strategy is that these strategies can be *measured* by examining development, cognition, and behavior, and then aggregating this information to “diagnose” an individual’s LH strategy. For an example, the mini-K uses indicators such as “I avoid taking risks,” “While growing up, I had a close and warm relationship with my biological mother,” and “I would rather have one than several sexual relationships at a time” to operationalize the latent K-factor (Table 1). Responses to such items are held to be (observable) manifestations of the unobservable LH strategy. Broader conceptualizations of traits related to LH strategy are proposed by super K-factors—such models forward additional variables (e.g., personality) to cluster with lower-level LH traits (e.g., Olderbak et al. 2014).

**Table 1:** The mini-K questionnaire (from Figueredo et al. 2006)

Item
1. I can often tell how things will turn out.
2. I try to understand how I got into a situation to figure out how to handle it.
3. I often find the bright side to a bad situation.
4. I don’t give up until I solve my problems.
5. I often make plans in advance.
6. I avoid taking risks.
7. While growing up, I had a close and warm relationship with my biological mother.
8. While growing up, I had a close and warm relationship with my biological father.
9. I have a close and warm relationship with my own children.
10. I have a close and warm romantic relationship with my sexual partner.
11. I would rather have one than several sexual relationships at a time.
12. I have to be closely attached to someone before I am comfortable having sex with them.
13. I am often in social contact with my blood relatives.
14. I often get emotional support and practical help from my blood relatives.
15. I often give emotional support and practical help to my blood relatives.
16. I am often in social contact with my friends.
17. I often get emotional support and practical help from my friends.
18. I often give emotional support and practical help to my friends.
19. I am closely connected to and involved in my community.
20. I am closely connected to and involved in my religion.

The present paper argues that current psychometric approaches to measuring LH strategies using self-report methods face conceptual problems. These conceptual issues render attempts to aggregate LH traits into K-factors a problematic practice, and ultimately of little theoretical worth. To put the thrust of this paper concisely: In order for LH strategy to be measured using a reflective latent variable measurement model, the item scores in the measurement instruments need to (at least in theoretical potential) represent reflections (i.e. effects) of a corresponding proximate mechanism. We demonstrate that K-factors do not meet this criterion for a reflective latent variable and thus do not succeed in measuring latent LH strategies. To arrive at this conclusion, the distinction between formative and reflective measurement models is reviewed, and we discuss the difference between “causal” and “effect” indicators. Second, the ultimate-proximate distinction in the evolutionary sciences and the position of LH strategies in this dichotomy will be discussed. We end this conceptual discussion by providing suggestions for new avenues in the psychometric approach to LH measurement.

## Reflective and Formative Constructs

Many psychological constructs cannot directly be observed and measured, and researchers in psychology consequently rely on indirect measurement instruments to quantify individuals’ position on such *latent* (unobservable) psychological variables (e.g., Bollen 2002; Borsboom 2008; Borsboom, Mellenbergh, and van Heerden 2003). The literature is full of variables that can be considered latent—such as personality and intelligence. These traits are not directly measured; instead researchers measure their reflections (e.g., associated behavior or utterances). LH strategy inventories, as measured using for instance the HKSS or mini-K scale*,* also assume (both explicitly and implicitly) the existence of a single latent variable involved in generating responses to the questionnaire items.

In principle, any measurement model of a latent variable used in psychological science can take one of two general forms (but see Bollen and Bauldry 2011). The standard measurement model in psychology is the reflective model (Bollen 2002; Borsboom et al. 2003), in which item-scores are seen to be *caused* by an underlying latent variable. It is one’s actual position on the unmeasured latent math skill variable that *causes* a particular answer to the question “Does two plus two equal four?” Similarly, social psychologists consider variables such as “attitudes” latent variables, measured by presumed reflections of the construct—for example, “Do you think object X is pleasant?” In standard reflective psychometric factor models, then, such items are modeled as “effect” indicators (Bollen and Bauldry 2011; Edwards and Bagozzi 2000), with the answers being *caused* by individuals’ position on the latent variable.

Recently, *formative* models have been recognized as an alternative method to capture constructs for which reflective models are conceptually inappropriate (e.g., Bollen and Bauldry 2011; Diamantopoulos et al. 2008; Diamantopoulos and Siguaw 2006; Edwards 2011; Jarvis et al. 2003). Item-scores in a formative model are seen to *create* a construct; in other words, the construct is a linear combination of the individual items. A clear example of this is socioeconomic status (SES); there is no underlying psychological process in individuals that corresponds to SES. Instead, SES is a social construct of interest to researchers and can be seen to meaningfully cluster individuals’ characteristics. In other words, unlike a trait such as intelligence, we cannot assume that SES exists independently of its measurement (see Borsboom et al. 2003). To clarify this using a metaphor, reflective models rely on logic of the form “the size of a fire can be estimated by the volume of the smoke,” but in formative models, indicators (e.g., educational background for SES) deliver the fuel determining the size of the fire. This difference in how observations are tied to their hypothesized constructs (as cause or effect) is what defines reflective versus formative models (Bollen and Bauldry 2011; Borsboom et al. 2003; Edwards and Bagozzi 2000).

**Fig. 1:**
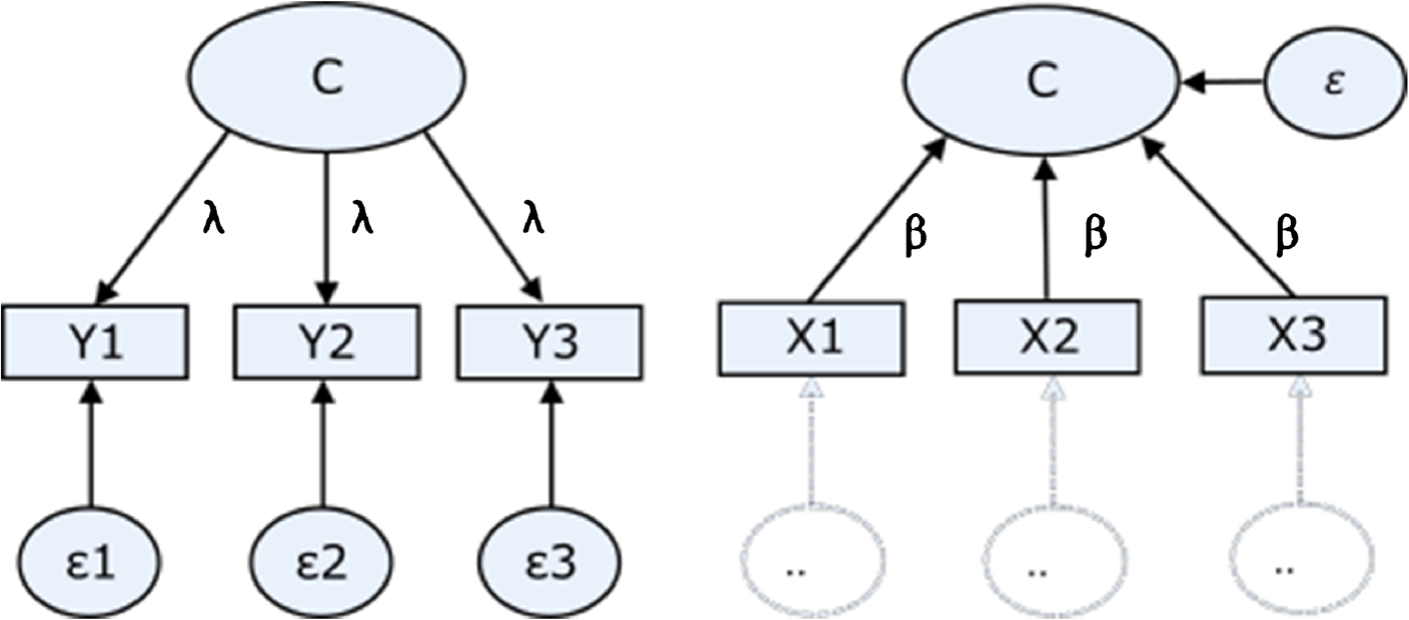
Example of a three-item reflective (left) and formative measurement model (right). In a reflective model (left panel), the psychological construct (C) is seen to cause item responses (Y) as a function of the respective factor loadings (**λ**). In this model, item-level measurement error (*ε)* can be estimated. The right panel depicts a formative construct, where the latent variable is composed as linear combination of the formative items. These items combine to form the construct as a function of their respective regression weights (**β**). In a formative model, only construct level measurement error can be estimated

Figure 1 depicts an example of a construct measured using the responses to three items. In panel A, the item scores are modeled as reflective indicators—an individual’s position on the latent variable is assumed to *cause* item responses. Panel B illustrates a formative model—the change of direction in the corresponding path (depicted with arrows) corresponds to the notion that causation now flows from indicator toward the construct. For instance, an individual’s response to questions assessing current income and education level *forms* this individual’s relative position on the SES construct; the information on such items linearly combines to create the construct.

Reflective models rely on a set of assumptions that need to be met to meaningfully use methods such as confirmatory factor analysis. First, latent variables rely on the principle of local independence, implying that given a particular latent variable, items are uncorrelated (e.g., Bollen 2002). This makes sense because when the latent variable is thought of as a common cause of item responses, taking this latent variable into account should (given measurement error) explain the correlations between these items. These assumptions, in turn, only make sense given an ontological assumption about latent variables: For latent variables to exert causal influence on item responses they need to *exist* in individuals’ psychology (Borsboom et al. 2003, 2004). If this ontological assumption does not hold for a given construct, then reflective models do not make much conceptual sense (Borsboom et al. 2003; see also Gruijters 2017). For example, the existence of a mechanism corresponding to intelligence is *hypothesized* to cause responses to items on an intelligence test, which explains why items become independent after conditioning the items on this common cause. Multiple indicators of a construct can thus only form a unidimensional scale given an ontological assumption about the latent variable (i.e., people actually possess a psychological mechanism *causally* involved in generating responses to questionnaires). Importantly, if the goal of psychological research is to uncover causal relationships between psychological variables and behavior, then researchers require the use of reflective measurement models that actually *measure* psychological mechanisms.

## The Measurement of Life History Strategy by K-factors

Current measurement of LH strategy (e.g., ALHB, mini-K, HKKS, and super K-factors) proceeds with reflective models, wherein scale items (or factors in the higher-order models) are considered to be *reflections* of an underlying latent LH strategy. This becomes evident from the factor models and internal consistency measures used to validate such scales (e.g., Figueredo et al. 2013).

Concerns and critiques about the assumptions underlying LH measurement models have been raised in the literature. Notably, Copping, Campbell, and Muncer (2014) used confirmatory factor analyses to investigate the unidimensionality of the HKSS (Giosan 2006). Despite testing multiple factor models, the researchers did not find any single factor model that fit the data well. Instead, their best-fitting model consisted of four correlated factors capturing conceptually distinct aspects of the presumed K-factor. Copping and colleagues have further argued that “the scales included in measures such as the ALHB . . . do not *assess* LH strategy as it is usually understood but rather represent variables that may predict or mediate LH trajectory” (2017: 2, emphasis added). Therefore, Copping et al. suggest that the utility of constructing overarching K-factors requires more consideration before sending such instruments to the “front lines” of LH research. Richardson et al. (2017b) elaborate and emphasize some of Copping et al.’s (2017) concerns by discussing at length the assumptions (ontological and causal) on which K-factors rely. Specifically, Richardson and colleagues argue that although LH researchers are not compelled to make such assumptions about their instrument, it is important to be aware that statistical procedures such as confirmatory factor analysis are inappropriate unless these assumptions are met.

While the existence of a latent variable implies that its manifestations become uncorrelated after taking this variable into account, reversal of this logic is not justified. That is, a well-fitting factor structure is a necessary, but not sufficient, condition for drawing ontological conclusions. Extending the fire-smoke metaphor, given that latent variables (fire) are causes of manifestations (smoke), it follows that the presence of fire implies smoke, but the presence of smoke does not imply the presence of fire. The incorrect inference that empirical evidence for the existence of discrete factors in the data equals ontological evidence for particular latent variables is salient in early K-factor research. Figueredo et al. (2006:139) concluded as much when arguing:

These results point to the existence of a single, highly heritable latent psychometric common factor (the K-Factor) that, as predicted by evolutionary ecological theory, underlies both the phenotypic and genetic covariances among a wide array of behavioral and cognitive life-history traits.

Empirical tests of hypothesized factor structures (for which confirmatory factor analysis would be the preferred method) work with an opposite logic than suggested in the citation. The conclusion allowed by factor analysis is that *n* factors describe the data (i.e., the variance-covariance matrix) to a certain extent, but whether these factors identify with latent variables cannot be concluded from factor analysis. Instead, hypotheses about underlying latent variables *justify* model specification in confirmatory factor analysis, or in the case of exploratory factor analysis, justify selection of the most meaningful factor structure. Empirical findings are thus not sufficient to assume the existence of latent variables; such hypotheses need to be deduced from theory. In many instances, whether it is feasible to hypothesize the existence of a latent variable (or psychological process) with a particular empirical factor can be determined a priori, because the merits of some hypotheses can be evaluated conceptually. We think that a reflective measurement model of the K-factor (such as the mini-K) can be ruled-out a priori because LH strategy (as measured by the K-factor) is an ultimate explanation and not proximate. Thus, modeling LH strategy with reflective indicators conflates the ultimate-proximate distinction in evolutionary theories of behavior; evolutionary theory does not justify the hypothesis that a single K-factor can describe LH strategy.

## Modeling Life-History Strategy as a Proximate Variable

The question of whether LH strategy can qualify as a reflective latent variable is complicated by the multiple levels of analyses evolutionary science involves in its research. To understand behavior, thought, and emotion, both ultimate and proximate explanations are required (Alessi 1992; Bateson and Laland 2013; Haig 2013; Laland et al. 2011; Mayr 1961, 1993; Scott-Phillips et al. 2011; Tinbergen 1963). Ultimate explanations, after further dividing Mayr’s dichotomy in Tinbergen’s (1963) categories, forward both functional and evolutionary explanations of behavior and respectively address the “what does it do?” and “how did it evolve?” questions about behavior (see also Bateson and Laland 2013). Phylogenetic histories of species are sometimes described as distal “causes” (e.g., Francis 1990), in the sense that species’ genomes have been adaptively shaped by natural selection, and natural selection can be seen as a cause for allele frequency changes in a population over time. Functional explanations, or Tinbergen’s subcategory of survival value, involve a “what does it do?” perspective. Functional statements say little to nothing about the causal mechanisms involved in behavior, although functional statements using fitness currency can be examined through, and perhaps exchanged for, proximate explanations.

Proximate explanations are those involved with “how does it work?” questions. They address questions about the ontogeny of traits, and the causal mechanisms in the “here and now” that produce behavior. From this it follows that to model human behavior by its *immediate causes*, only references to Tinbergen’s causation category (the mechanisms) are valid—since by definition, ultimate explanations do not address the mechanisms producing behavior. Psychometrics is a discipline that attempts to measure individuals’ “here and now” psychology by statistically connecting overt behavior and utterances to proposed underlying latent variables. Therefore, given our discussion on the nature of reflective models and their underlying ontological assumption, latent variables can only be hypothesized at the proximate level.

LH strategies, then, provide ultimate explanations of particular traits and explain why traits cluster by referring to fitness effects. Figueredo et al. (2004) were explicit in describing the K-factor as providing functional-level explanations: “LH theory suggests that natural and sexual selection will combine LH traits into functional composites representing co-adapted reproductive strategies” (2004:123). Indeed, reference to a particular LH strategy allows us to better understand why particular behaviors cluster together, they add meaning to our understanding of the proximate mechanisms involved in behavior. In the above citation, the authors quite adequately describe the K-factor as a functional composite, not as a proximate mechanism that could fulfill the requirements of reflective measurement. This raises the question of what information scores on K-factors are conveying. That is, when researchers compute a factor score of a latent variable that is not reflective of a proximate mechanism, then what does this represent? Put bluntly, K-factor scales do not meet the (causal and ontological) criteria for test validity as submitted for instance by Borsboom et al. (2004:1067): “The concept of validity . . . expresses nothing less but also nothing more than that an attribute, designated by a theoretical term like intelligence [or, LH strategy], exists and that measurement of this attribute can be performed with a given test because the test scores are causally affected by variation in the attribute.” Although the reflective approach to assessing functional descriptions is unwarranted and K-factors are not actually *measuring* LH strategies, there are alternative models relying on assumptions that could be satisfied by K-factor scales.

## An Alternative Approach: Formative Models

**Fig. 2:**
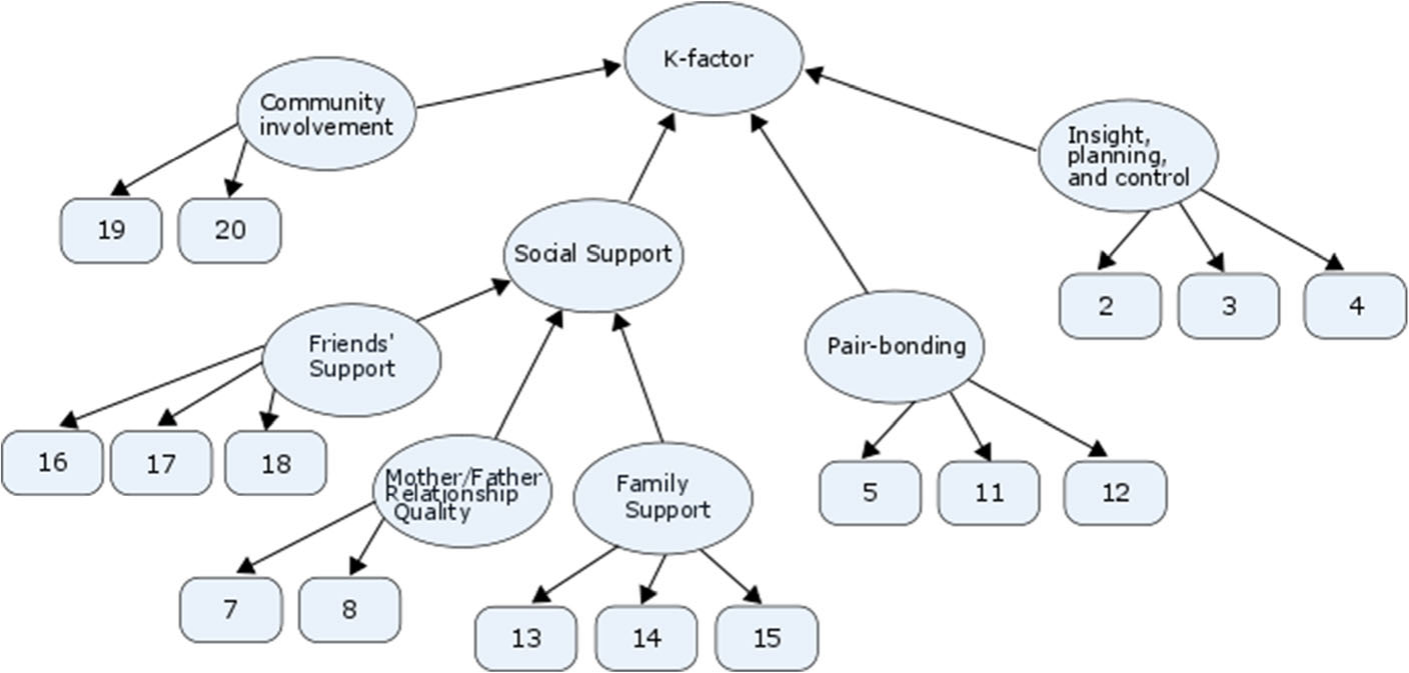
A proposed formative model creating a K-factor index based on Richardson et al. (2017a). Path directionality indicates whether indicators are causes (formative) or effects (reflective) of their respective construct. Cross-loadings have been omitted for graphical clarity. Item numbers correspond to the mini-K content depicted in Table 1

To deflate the proximate-ultimate distinction in K-factor models, we suggest that LH strategy could be modeled as a formative construct, one that is *descriptive* of an individual, similar to SES. The items in the mini-K (and related measurement instruments) should, in our view, be modeled as reflections of various proximate constructs. Subsequently, these constructs can be used to construct a formative measurement model. Figure 2 depicts a proposed formative measurement model for the mini-K, based on recent findings by Richardson, Chen, Dai, Brubaker, and Nedelec (2017a). LH strategy modeled as a formative construct meaningfully clusters various proximate mechanisms to allow them to be collectively informative about individuals. Such a formative model aligns more closely with the ultimate-proximate distinction for behavioral explanations.

We propose the following strategy for future LH research using a psychometric approach. First, validate how proposed proximate mechanisms of LH strategy regress on a functionally defined K-factor using formative measurement models and assess model fit with the data. Second, do not create composites of an underlying K-factor (which, as we have argued, would be tantamount to defining functional descriptions at the proximate level), but only use first-order constructs that are hypothesized to identify with discrete proximate mechanisms (cf. Edwards 2011). These proximately defined constructs (and resulting composites) can subsequently be meaningfully modeled as predictors in regression models, path models, or structural equation models.

Importantly, specifying LH strategy as formative rather than reflective is not merely conceptually apt, but the models require different analytical strategies—and thus this decision has potential statistical consequences. As discussed by Diamantopoulos, Riefler, and Roth (2008), incorrectly modeled indicator-construct relationships can lead to incorrect conclusions about structural relationships among variables, as well as biased estimates of model fit. For example, Law and Wong (1999) demonstrate that misspecification of a formative construct as reflective leads to overestimation of the effect of the misspecified variable on an outcome variable. Jarvis, MacKenzie, and Podsakoff (2003) replicate these findings and also show that regression coefficients of predictor variables are underestimated when a formative outcome variable is wrongly modeled as reflective. Similarly, estimations of model fit yield biased indices as well (Diamantopoulos and Siguaw 2006; Edwards 2001; Jarvis et al. 2003).

Indeed, the reflective-formative distinction is critical to psychometric practice more broadly, including but not limited to scale reliability analysis and various forms of factor analysis (see Bollen and Diamantopoulos 2017). The notion that a formative construct incorrectly specified as reflective violates the assumptions of common psychometric procedures, and might lead to biased empirical conclusions in structural models, underlines the relevance of clearly conceptualizing LH strategy.

## Discussion

We have argued that because a discrete proximate mechanism corresponding to LH strategy (such as the K-factor) cannot be assumed, current approaches do not succeed in measuring reflections of such a latent variable. Current psychometric measurement of LH strategy involves an unwarranted conflation of functional (i.e., ultimate) descriptions and proximate mechanisms—a conceptual mix-up that may generate unviable hypotheses and invites misinterpretation of empirical findings. Thus, common psychometric measurement instruments of LH strategy (including ALHB, mini-K, and HKKS) incorrectly assume reflective measurement models implying that each individual proximate mechanism is conceptually equivalent and, by extension, is a (locally) independent measurement of the latent LH strategy.

We thus suggest a different approach to LH measurement, one that treats K-factors as *formative constructs*—giving a meaningful summary of an individual’s characteristics, akin to how SES is conceptualized. In doing so, LH strategy becomes a descriptive construct, giving a meaningful description (rather than causal explanation) for the observed correlations between LH traits. Acknowledging K-factors as formative (and as ultimate) prevents conceptual errors, such as inappropriate causal inferences and inappropriate extensions of empirical findings toward theory development. The use of such a formative measurement model, as discussed, has direct consequences for the parameter estimates in regression models including a psychometric measurement of the K-factor.

Although we have shown conceptual problems in considering K-factors *measures* of LH strategy, we are not disputing that LH strategy theoretically *could* correspond to a discrete proximate mechanism. Additionally, our concerns with the psychometric approach do not extend to developmental approaches aiming to explain variation in LH strategies (e.g., Belsky et al. 1991; Del Giudice 2009). However, we leave open the empirical question of whether variation in the bundle of proximate mechanisms captured by K-scales is further reducible to a single proximate mechanism (e.g., impulsivity or reward-sensitivity; Frankenhuis et al. 2016) with which the K-factor scores could identify. If such a reduction is not possible, researchers need to examine hypotheses about these mechanisms separately (as we have suggested). If reduction to a single mechanism is possible, creating latent variable models of LH strategy would be feasible, but it would need to measure LH strategy through direct causal manifestations of such a mechanism. Nonetheless, analyses of K-factor instruments by Copping et al. (2014) and Richardson et al. (2017a), also cast doubt on the possibility that K-factor scores identify with a latent LH strategy (see also Copping et al. 2017; Richardson et al. 2017b).

What are other possible new directions for the psychometrics of LH strategies? The discussion thus far may suggest that the K-factor’s conceptual problems are solved by a simple reversal of path directionality in factor analytic models—turning reflective measurement models into the formative kind. Although this might be a step LH research needs to take to conceptually align theory with measurement, the value of such descriptive constructs in psychological research is far from clear. In particular, can a descriptive construct (summarizing various conceptually different sources of information about individuals) be fruitfully used as a predictor of behavioral outcomes? For psychologists interested in examining psychological constructs as causal antecedents of behavior to further psychological theory, such descriptive variables may be of little theoretical value. This is simply because such constructs are not *measurements of* the mind, but rather *describe* individuals’ psychology. As Richardson et al. (2017b) note, such a descriptivist approach “can be seen as more concerned with statistical parsimony than elucidating the nature of causal forces responsible for patterns of covariation” (2017b:2). Other scholars (e.g., Rhemtulla et al. 2015), for this reason, have advocated that formative models using causal indicators of constructs should best not be described as “measurement models.”

Although these suggestions conceptually align LH measurement with common psychometric approaches, there is (as the authors announce) a “new psychometric game in town”: the network approach to psychometrics (e.g., Borsboom and Cramer 2013; Epskamp et al. 2016) that we suggest as a direction for future research. Network models do not assume that constructs merely exist by virtue of their operationalization (as formative models), but they also do not assume that latent variables exist as a single “entity” or mechanism (as reflective models). Instead, network models take an intermediate ontological position; they define constructs in terms of a network of multiple interacting manifestations. This approach might prove to be of particular value to researchers interested in modeling *functionally defined* constructs, such as LH strategy, because functions may very well be examined through a network of proximate mechanisms. In such a model, LH strategy could be depicted as a network of proximate mechanisms that, as a whole, defines a person’s LH strategy.

In conclusion, the conflation of ultimate and proximate causes in the evolutionary behavioral sciences is not a problem unique to LH measurement (for a discussion, see Scott-Phillips et al. 2011). Proximate models of behavior lose their conceptual clarity—and indeed their causal explanatory potential—when ultimate factors referring to fitness effects are included. Mayr and Tinbergen’s effort to distinguish between ultimate and proximate causation remains important in evolutionary behavioral science—also for the quality and validity of measurement instruments. How to properly use ultimate explanations in proximate empirical models remains an important issue for progress in evolutionary behavioral science. In our view, the specification of formative measurement models when describing and testing functionally defined constructs might be a first important conceptual step.
